# Supplementary material for: Recurrence rates after surgical removal of oral leukoplakia—A prospective longitudinal multi-centre study
Source: PLoS One. 2019 Dec 6;14(12):e0225682. doi: 10.1371/journal.pone.0225682 (PMC6897554; doi:10.1371/journal.pone.0225682)
Supplement: Attachment — Submitted filename: Respons to the Reviewers.docx [file pone.0225682.s001.docx]

**PLOS ONE**

**Academic Editor**

Dr. Muy-Teck Teh

Gothenburg, 2019 October 31^th^.

Dear Dr Muy-Teck Teh

Thank you very much for the thorough revision of our manuscript entitled *“Recurrence rates after surgical removal of oral leukoplakia - a prospective longitudinal multicentre study” (*PONE-D-19-26374*)*, which is highly appreciated.

We have amended the manuscript and done changes in the manuscript in accordance with suggestions from the referees and from the editors. We have responded (*in italics*) to your comments and the referees´ questions item by item (please see below).

In the revised version, we have addressed the following requirements that the journal have addressed:

1. Please ensure that your manuscript meets PLOS ONE's style requirements, including those for file naming.

*We have checked the manuscript against the style requirements.*

2. Please include the full name of the IRB/ethics committee that reviewed and approved this study, including the name of the affiliated institution if applicable. We additionally ask that you include your IRB/ethics committee approval number in your ethics statement.

*Amended P. 6, para 3: “The study was approved by the Regional Ethical Review Board in Gothenburg, Sweden (Dnr. 673-10)”*

3. We suggest you thoroughly **copyedit your Abstract** for language usage, spelling, and grammar. If you do not know anyone who can help you do this, you may wish to consider employing a professional scientific editing service

*The manuscript including the Abstract has been proofread by a professional scientific editor.*

4. Please provide additional details regarding participant consent in the Methods section of your manuscript. Please ensure that you have specified (1) whether consent was informed and (2) what type you obtained (for instance, written or verbal, and if verbal, how it was documented and witnessed).
   If your study included minors, state whether you obtained consent from parents or guardians.

*Amendment in the revised manuscript P.6, Para.3:*

*“All patients were given both written and verbal information about the study's objectives. Both a written and a verbal consent was obtained from the patients at the clinic before the inclusion. The written consent was signed by the patient and the clinician that included the patient in the study. The study was approved by the Regional Ethical Review Board in Gothenburg, Sweden (Dnr. 673-10) and was conducted in accordance with the Helsinki Declaration.”*

*We did not study minors.*

5. In your Methods section, please provide additional information about the participant recruitment method and the demographic details of your participants. Please ensure you have provided sufficient details to replicate the analyses such as: a) the recruitment date range (month and year), e) a description of how participants were recruited, and f) descriptions of where participants were recruited and where the research took place (institution names of the participating centres).

*Amendment in the revised manuscript*

*a) Amended: P.5, Para.1*

*e) Amended: P.5, Para.1*

*f) Amended: P.4, Para.4*

6. We note that you have indicated that data from this study are available upon request. PLOS only allows data to be available upon request if there are legal or ethical restrictions on sharing data publicly. For information on unacceptable data access restrictions, please see <http://journals.plos.org/plosone/s/data-availability#loc-unacceptable-data-access-restrictions>.

*There are ethical and legal restrictions on sharing our data set according to Swedish law. Study data contain sensitive patient information that can be connected to individual patients. It may be possible to identify and connect personal information from the data set, even though data is de-identified.*

*All relevant data within the manuscript are at group level. Data cannot be shared publicly because of that all data are research data and belongs to University of Gothenburg. Data are available upon request based on ethical and legal restrictions from GU server, University of Gothenburg. Institutional Data Access Contact: Associate Professor Bengt Hasséus, Department of Oral medicine and Pathology, Institute of Odontology, the Sahlgrenska Academy, University of Gothenburg, Sweden at* [*bengt.hasseus@gu.se*](mailto:bengt.hasseus@gu.se) *or the Head of the Institute of Odontology, Professor Peter Lingström at peter.lingstrom@odontologi.gu.se for researchers who meet the criteria for access to confidential data.*

**Response to the reviewers´ comments,**

Thank you very much for valuable comments. We are much obliged for your thorough revision. Please find our response in italics below.

Please find a point-by-point response to the Reviewers comments below.

Reviewer # 1.

“Oral leukoplakia with dysplasia are potentially premalignant lesions. In the current study the number of cases with dysplasias that is selected are only 22. More number of cases with dysplasias can be evaluated and the recurrence rate and causes for recurrences for these cases should be determined. This will help in identifying high-risk lesions and they can be treated appropriately.”

*We agree. But the 22 cases with dysplasia are the patients to date available in this prospective study. However, the ORA-LEU-CAN study is ongoing and we hope in the future to be able to answer the issues raised by the referee.*

Reviewer #2.

1) “A definition of oral leukoplakia is given in the introduction, however, I think the definition used by the participating centers should be given also in the patients and methods section#.

*The definition of OL has been included in the Patients and Methods section (P.5 para.1).*

2) “…the inclusion criterion was a clinically and histopathologically verified diagnosis of OL” is correct.

*We agree that OL is a solely clinical diagnosis. The sentence has been rephrased:
“the inclusion criterion was a clinically verified diagnosis of OL” (p.5, para. 1).*

3) In Table 1 lichenoid reaction is given as the histopathological diagnosis in 20 cases.

*Table 1 illustrates the patients’ characteristics in the entire patient group (N = 180) but we analysed only the 103 patients who had an OL that we were able to be completely remove. Of these seven patients four recurred.*

Three questions emerge from this*:*

a. Is this compatible with a final diagnosis of leukoplakia? A short discussion could be included.

*We have elucidated this matter in the Discussion (p.12, para.2):*

*“In seven patients with OL the histopathological diagnosis was lichenoid reaction. In these patients, records and clinical photos were thoroughly re-reviewed. No clinical signs according to van der Meij et al (26), were found indicating oral lichen planus, oral lichenoid lesion or lichenoid contact reaction. A diagnosis of OL according to WHO (1) postulates exclusion of known diseases or disorders that carry no increased risk of cancer. In the present study we as strictly adhered to this definition. In the WHO definition of OL, histopathological diagnosis is confined to presence of dysplasia or not (1). Thus, excluding known diseases causing a white plaque in the oral mucosa, the histopathological diagnosis lichenoid reaction is compatible with an OL diagnosis.”*

b. Lichenoid reactions are sometimes seen in relation to contact with dental restorations or other causes. Were there any clinical indications that the cases with histopathologically diagnosed lichenoid reactions had an obvious (or possible) cause such as contact with dental restorations? I think a short mentioning of this should appear in the patients and methods section.

*None of the seven patients had dental fillings in contact with the lesions. A sentence describing this has been added in the manuscript (P.x, para y) In that group seven patients had the histopathological diagnosis lichenoid reaction.*

c. How many recurrences were seen in these cases

   *4 OL out of 7 with the histopathological diagnosis lichenoid reaction recurred.*

On behalf of the authors,

Yours sincerely,

Bengt Hasséus, LDS, PhD, Associate Professor

Corresponding author

Dept. of Oral Medicine and Pathology,

Institute of Odontology, Sahlgrenska Academy,

University of Gothenburg,

PO Box 450,

SE 405 30 Gothenburg, Sweden

Email: [bengt.hasseus@gu.se](mailto:bengt.hasseus@gu.se)
